# Research progress of ferroptosis pathway and its related molecular ubiquitination modification in liver cancer

**DOI:** 10.3389/fonc.2025.1502673

**Published:** 2025-03-21

**Authors:** Silin Yao, Yi Quan

**Affiliations:** ^1^ The First Clinical Medical School, Guangdong Medical University, Zhanjiang, Guangdong, China; ^2^ The First People’s Hospital of Zhaoqing, Guangdong Medical University, Zhaoqing, Guangdong, China

**Keywords:** liver cancer, ubiquitination, ferroptosis, lipid peroxidation, targeted therapy

## Abstract

As a new type of programmed cell death, ferroptosis is characterized by iron metabolism disorder and reactive oxygen species (ROS) accumulation, and is involved in regulating the occurrence and development of cancer cells. Especially in the field of liver cancer treatment, ferroptosis shows great potential because it can induce tumor cell death. Ubiquitination is a process of protein post-translational modification, which can affect the stability of proteins and regulate the progress of ferroptosis. This article reviews the research progress of ubiquitination modification of molecules related to ferroptosis pathway in the regulation of liver cancer, providing a new strategy for the treatment of liver cancer.

## 1 Introduction

Hepatocellular carcinoma (HCC) is one of the most common malignant tumors, accounting for an extremely high proportion of all cases of primary liver cancer, about 75% to 85%. It has become a major problem in the field of global health, and has brought major tests and challenges to the public health system (1). Although there are many methods for the treatment of liver cancer, the drug resistance and prognosis of patients still need to be urgently improved. Therefore, it is a problem to find a new targeted treatment for liver cancer.

Ubiquitin is a small molecule protein composed of 76 amino acids (2). Ubiquitination, defined as covalent binding of ubiquitin small molecule proteins to target proteins, is one of the most common post-translational modifications in proteins. Ubiquitination is a multifunctional intracellular regulatory mechanism that affects the fate and function of proteins by labeling them, thus playing a central role in maintaining the health of cells and organisms. This is a finely regulated biochemical process, which is completed by the coordinated action of three key enzymes - E1 (ubiquitin activating enzyme), E2 (ubiquitin conjugating enzyme) and E3 (ubiquitin ligase) (3). Studies have found that ubiquitination is closely related to the stability of oncogenes and tumor suppressor genes (4, 5). This indicates that we can find new targeted therapies by exploring the effects of ubiquitination on tumors.

In the development of life, cell death is an important link in both physiological and pathological conditions, indicating the end of cell life. According to whether cell death is regulated by genes or various signaling pathways, the known types of cell death include apoptosis, necroptosis, pyroptosis, ferroptosis, and autophagy (6). And cuproptosis proposed by Peter Tsvetkov et al. in 2022 (7) and disulfidptosis proposed by Zheng t et al. in 2023 (8). Among them, ferroptosis, a previously unreported mode of programmed cell death, was first proposed by Dixon et al. in 2012 (9). Each cell death mode has its specific molecular mechanism and biological function, and plays an important role in maintaining the homeostasis of organisms and coping with various pathological conditions. Existing studies have shown that ferroptosis is associated with excessive iron accumulation and lipid peroxidation. When the amount of intracellular iron ions exceeds the capacity of the cell, it will react with polyunsaturated fatty acids (PUFAs) on the cell membrane to produce a large amount of reactive oxygen species (ROS), causing damage to various organelles and ultimately leading to cell death (10) As a newly discovered programmed oxidative damage, compared with pyroptosis (11), necrosis (12), and autophagy (13), the signaling pathways, cell morphological characteristics, inducers, and inhibitors that mediate the initiation and progression of ferroptosis are very different. The obvious cell morphological sign of ferroptosis is the shrinkage of mitochondrial volume, and the outer membrane may rupture. Moreover, the number of cristae in mitochondria decreased, sometimes even completely disappeared. Nevertheless, the morphology of the nucleus seems to remain relatively stable during this process without very obvious changes. This form of organelle change is an important feature of ferroptosis, which is different from other cell death modes (14).

However, the research on the post-translational regulation of ferroptosis related proteins at home and abroad is not deep enough. Several recent studies have shown the role of ubiquitination system and its complex in regulating the core regulatory factors of ferroptosis, which mediates ferroptosis of tumor cells and has an important impact on enhancing the sensitivity of cell death (15). In conclusion, with the rapid development of genomics and proteomics, exploring the role of ubiquitination system in the regulation of ferroptosis may help to develop new therapeutic strategies for liver cancer.

## 2 Ferroptosis pathway

**Figure 1:**
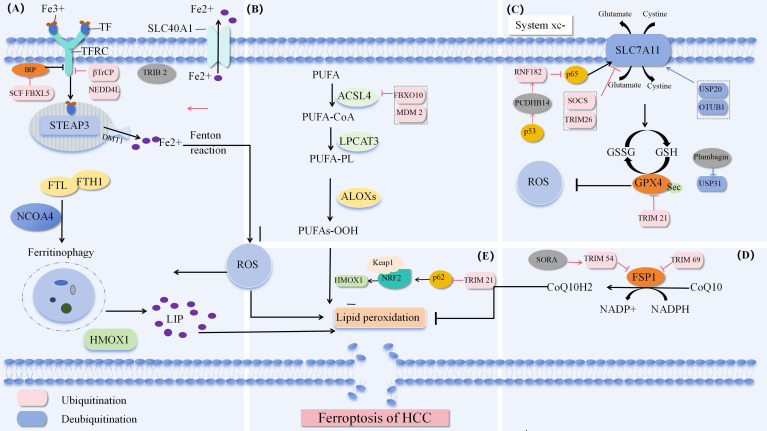
Ferroptosis pathway **(A)** Iron metabolism pathway. **(B)** Synthesis and peroxidation of unsaturated phosphate. **(C)** System Xc-. **(D)** NADPH-FSP 1-CoQ Defense pathway. **(E)** P62-Keap 1-NRF 2 (84).

Ferroptosis is a form of cell death, and it is generally believed that iron and lipid peroxides are the key factors in the process of ferroptosis. This process involves a variety of regulatory mechanisms, mainly including iron metabolism pathway, cystine glutamate antiporter pathway (System Xc-) and lipid peroxide accumulation pathway. Moreover, erastin induces ferroptosis by inhibiting the voltage dependent anion channel (VDAC) of mitochondria. The latest research found that NADPH-FSP 1-CoQ pathway, as an independent system, works synergistically with glutathione peroxidase 4 (GPX 4) and glutathione (GSH) to inhibit lipid peroxidation **Figure 1**.

### 2.1 Iron metabolism pathway

Iron is an essential trace element in the life process of organisms. It exists in the form of ferric (Fe3+) and ferrous (Fe2+) ions and participates in redox reactions. For cells, the lack or accumulation of iron will lead to the destruction of redox homeostasis, causing cell death, including ferroptosis, so regulating iron homeostasis is extremely important. Fe3+ is transported in the blood circulation after binding with transferrin (TF) and enters cells through transferrin receptor (TFRC/TfR 1) -mediated endocytosis. Subsequently, Fe3+ is separated from TF, converted to Fe2+ by STEAP 3 metal reductase redox reaction, and then transported to the cytosol by DMT-1 (SLC 11A2, solute carrier family 11 member 2). Unbound cytosolic Fe2+ can be transported to mitochondria or exist in the form of labile iron pool (LIP), while excess iron is stored in ferritin (16). Through in-depth analysis of themechanism of iron metabolism, we can know that the whole metabolic cycle of iron element has an impact on the susceptibility to ferroptosis, and the synthesis and degradation process of ferritin is closely linked to the storage and release process of iron ions in cells. In addition, by regulating nuclear receptor coactivator 4 (NCOA 4) - mediated ferritin autophagy, cells are able to finely regulate the availability of iron ions to support their metabolic needs and prevent potential toxicity caused by iron overload (17, 18). The export of intracellular excess Fe2+ across the cell membrane requires the help of ferroportin (FPN/SLC40A1) to export across the membrane to the outside of the cell to maintain iron homeostasis and reduce ferroptosis. Because Fe2 + is characterized by instability and high reactivity, Fe2+ accumulates and generates hydroxyl radicals (· OH) through Fenton reaction. These highly reactive free radicals rapidly react with polyunsaturated fatty acids (PUFAs) in cell membranes and organelle membranes to form a large amount of reactive oxygen ROS, and oxidative stress causes ferroptosis.

Studies have shown that increasing iron uptake through TFRC overexpression, or reducing iron storage through ferritin knockdown, inducing ferritin autophagy, and reducing cellular iron export through knockdown of FPN ultimately enhance sensitivity to ferroptosis (17). The RNF217gene is a target of Tet1 and uses its E3 ubiquitin ligase activity to regulate the ubiquitination and subsequent degradation of FPN (19). In addition, iron ions not only mediate the generation of oxidative stress, but also participate in regulating the activity of antioxidant enzymes. Iron ions can activate or inhibit the activity of glutathione peroxidase (GPX 4). When GPX 4 activity is inhibited, the intracellular lipid ROS level will rise sharply, leading to cell membrane damage and cell death. On the contrary, if these antioxidant enzymes can remain active, they can help remove excess lipid ROS and protect cells from ferroptosis. Therefore, exploring the specific molecular mechanism between iron ions and GPX 4 is helpful to regulate the sensitivity of liver cancer to ferroptosis.

### 2.2 Synthesis and peroxidation of unsaturated phosphate

Free polyunsaturated fatty acids (PUFA) are linked to COA via acetyl CoA synthetase long chain family 4 (ACSL 4) to generate corresponding acyl CoA derivatives. PUFA-CoA is re-esterified in phospholipids via lysophosphatidylcholine transferase 3 (LPCAT 3) to generate polyunsaturated phosphate PUFA-PLs. ACSL 4 promotes the synthesis of PUFA phosphatidylethanolamine (PUFA-PE in the cell membrane, and subsequently lipoxygenase (LOX) directly catalyzes PUFAs, resulting in the accumulation of ROS causing oxidative stress and loss of structural integrity of biological membranes (20). Therefore, ACSL 4 and LPCAT 3 can enhance the production of polyunsaturated phosphate and promote ferroptosis. Liao et al. found that CD8+ T cell-derived interferon γ (IFN γ) can target the promoter region of ACSL 4 and transcriptionally stimulate the expression of ACSL 4 in tumor cells. It can also combine with PUFA to become a natural ferroptosis inducer (FIN), and stimulate tumor ferroptosis by changing the lipidomic pattern of tumor cells through ACSL 4 (21). Other studies have shown that solute carrier family 7 member 11 (SLC 7A 11) and solute carrier family 3 member 2 (SLC 3A 2) are inhibited by interferon gamma (IFN γ) released from CD 8+T cells, thereby inhibiting cystine uptake by tumor cells, reducing the activity of GPX 4, and activating the process of lipid peroxidation in tumor cells (22). In addition, miR-552-5p reduces the sensitivity of HCC to ferroptosis by targeting the 3 ′ UTR of ACSL 4, downregulating the expression level of ACSL 4, and inhibiting the synthesis of PUFA-PE (23). It can be seen that ACSL 4 is of great significance for the development of ferroptosis. The latest study found thatPC-PUFA2, a phospholipid with two polyunsaturated fatty acyl tails, interacts with the mitochondrial electron transport chain to produce reactive oxygen species (ROS), induce lipid peroxidation, and promote ferroptosis (24). In addition, peroxidation of n-3 and n-6 polyunsaturated fatty acids (PUFAs) selectively induces ferroptosis in an acidic tumor environment (25). It is worth noting that GPX 4 and ACSL 4 play opposite roles in the regulation of ferroptosis. GPX 4 inhibits ferroptosis by inhibiting lipid peroxidation, while ACSL 4 promotes ferroptosis by promoting lipid peroxidation.

### 2.3 System Xc- pathway

The current study revealed that the System Xc-/GSH/GPX 4 axis is widely recognized as an important antioxidant system (26). System Xc- is a glutamate cystine antiporter on the cell membrane. It consists of two subunits, SLC 7A 11 and SLC 3A 2, which form a heterodimer. It depends on the higher concentration of glutamate in the cell to drive the uptake of lower concentration of cystine outside the cell. Cystine enters the cell and is reduced to cysteine to provide raw material for GSH synthesis. GSH is an important cofactor in the activation of GPX 4.GPX 4 catalyzes the reduction reaction of lipid peroxides, converts harmful lipid peroxides into corresponding alcohol compounds, and prevents the formation of Fe2+ dependent ROS, thus protecting the cell membrane from oxidative damage and maintaining the integrity of the membrane (27). Therefore, maintaining a certain level of GSH is of great significance for affecting the activity of GPX 4 and regulating ferroptosis. The study found that the use of inhibitors or inducers of ferroptosis triggered by System Xc- to interfere with tumor cells showed good effects on tumor treatment. Among them, erastin, the first type of ferroptosis inducer, directly inhibits System Xc-, leads to the reduction of cystine absorption, affects the synthesis of GSH, indirectly reduces the activity of GPX 4, destroys redox homeostasis and accelerates the aggregation of ROS, resulting in the decline of cellular antioxidant capacity, and finally promotes the occurrence of ferroptosis (28). In addition, erastin can also regulate ferroptosis through VDAC and p53 (29).

However, it is interesting to note that RSL 3, a second ferroptosis inducer, did not lead to a decrease in the level of intracellular GSH, but directly inhibited the activity of GPX 4, triggering ferroptosis (30). Therefore, it is reasonable to speculate that the activity of GPX 4 is the key to the regulation of ferroptosis process by system xc-. In addition, although the exact mechanism of how erastin inhibits System Xc- has not been fully elucidated, studies have shown that it may be closely related to the expression or function of SLC 7A 11 (31).

### 2.4 NADPH-FSP 1-CoQ Defense pathway

Studies have shown that inhibition of GPX 4 in some cells does not induce ferroptosis, but that another regulatory molecule, ferroptosis inhibitory protein 1 (FSP 1), acts independently of glutathione (32, 33). FSP 1 functions by activating the metabolic pathways of coenzyme Q10 (CoQ 10) and NADPH. FSP 1 consumes NADPH and reduces coenzyme Q10 to coenzyme Q10 H2. Coenzyme Q10 H2 is a lipophilic antioxidant that prevents lipid peroxidation and inhibits ferroptosis by trapping free radicals. In addition, FSP 1 is also involved in the unconventional redox cycle of vitamin K. In this process, FSP 1 acts as vitamin K reductase (VKR), which uses NADPH as an electron donor to convert vitamin K to its reduced form, VKH2. As an antioxidant, VKH2 can effectively trap free radicals, thereby inhibiting the lipid peroxidation process and playing an anti ferroptosis role (34).

### 2.5 Other ways of ferroptosis

In 2015, sunxiaofang’s team constructed a model of HePa 1-6 cells in mice with knockdown of nuclear factor erythroid 2-related factor 2 (NRF 2), and performed Q-PCR analysis. It showed that knockdown of NRF 2 increased the occurrence of ferroptosis in HCC, and outlined a new signaling pathway mediating ferroptosis: p62- keap 1-NRF 2 pathway (35). However, some studies showed that the effect of p62 on tumor growth was completely abolished by knocking down NRF 2, indicating that NRF 2 activation via p62 phosphorylation promoted the proliferation of HCC (36). It can be seen that the specific mechanism of p62- keap 1-NRF 2 pathway in HCC still needs to be further studied. It has been reported that when cells are under oxidative stress, the essential amino acid methionine is converted to cystine through the sulfur transfer pathway, which assists in the synthesis of GSH and plays an anti ferroptosis role through the GPX 4 pathway (37). In addition, ATG 5-ATG 7-NCOA 4 (38) and glutamine metabolic pathways (39) and Sigma-1 receptor (S1Rs) pathways (40) cooperate through their respective unique mechanisms of action and signaling pathways to jointly fine regulate intracellular iron metabolism and ROS balance, maintain the stability of intracellular environment and resist oxidative stress. Further study found that p53 inhibition of SLC 7A 11 expression would lead to the decrease of intracellular GSH level, which in turn affected the activity of GPx4, leading to the reduction of antioxidant capacity, ROS accumulation and ferroptosis (41). The p53-SAT 1-ALOX 15 pathway is also involved in affecting ferroptosis (42). Interestingly, under certain conditions, p53 inhibits the cell cycle by inducing the expression of the cell cycle regulatory protein p21, thereby converting part of serine used to synthesize nucleic acids into GSH, which helps to inhibit the occurrence of ferroptosis (43). In conclusion, p53 can both promote and inhibit the occurrence of ferroptosis, and its specific mechanism of action in tumor treatment needs to be further improved.

## 3 Modification of ubiquitination of ferroptosis pathway related molecules

### 3.1 Ubiquitination of TFRC

Intracellular transferrin receptor (TFRC), ferroportin (FPN, also known as hepcidin) and iron binding protein (FN, ferritin) are all post transcriptionally regulated by iron regulatory protein (IRP), which regulates the level of protein by binding or dissociating with iron response element (IRE) on mRNA. Ubiquitin ligases can recognize and ubiquitinate two IRPs with ire binding activity, reduce IRP protein levels, lead to iron homeostasis imbalance, and then induce cell ferroptosis (44).

TFRC is a protein expressed on the cell surface. By binding to transferrin in blood, it transports iron ions (Fe² +) into cells, participating in maintaining iron homeostasis and affecting the sensitivity of ferroptosis (45). Tribbles homolog 2 (TRIB 2) has been well demonstrated to promote liver tumorigenesis by regulating E3 ligase in the ubiquitin proteasome system (UPS) (46). There is evidence that TRIB 2 promotes TFRC ubiquitination and ultimately reduces labile iron in liver cancer cells by interacting with E3 β TrCP. Deletion of TRIB 2 leads to the opposite result. TRIB 2 is involved in regulating RSL 3 and erastin induced ferroptosis (47). In addition, TRIB 2 reduced the ubiquitination of GPX 4 by reducing the stability of ubiquitin, thereby slowing down the degradation rate of GPX 4. This mechanism helps to maintain the stability and activity of GPX 4, thereby enhancing the antioxidant capacity of cells and alleviating the effect of oxidative damage in liver cancer cells (48). Notably, TRIB 2 inhibits Wnt signaling activity by promoting the ubiquitination of its key factors TCF 4 and β -Catenin in liver cancer cells, thereby inhibiting tumor growth (49). However, whether the process involves ferroptosis of liver cancer cells still needs further study. Studies have shown that ubiquitination of upstream molecules can indirectly regulate TFRC, thereby affecting the sensitivity of ferroptosis. In a mouse stroke model, overexpression of methyltransferase like protein 3 (L3) upregulated the expression of the E3 ubiquitin ligase NEDD 4L by methylating and stabilizingNEDD 4L mRNA, which binds to TFRC and mediates its ubiquitination and degradation, reducing iron accumulation and inhibiting the iron dependent oxidative cell death pathway (50).

### 3.2 Ubiquitination of ACSL 4

Regulating the expression level of ACSL 4 is of great significance for affecting the sensitivity of ferroptosis in HCC. Researchers have been actively exploring the mechanism affecting the expression level of ACSL 4. As early as 2014, studies have shown that arachidonic acid (AA) downregulates the expression level of ACSL 4 in hepatocytes by promoting ACSL 4 ubiquitination and proteasomal degradation (51). With more in-depth exploration, chencongcongcong’s team demonstrated for the first time that the overexpression of cytochrome P450 1B 1 (CYP 1B 1), a metabolic enzyme of arachidonic acid metabolic pathway, led to its metabolite 20-HETE to induce the expression of E3 ubiquitin ligase FBXO 10 by activating PKC signaling, thereby increasing the ubiquitination and degradation of ACSL 4, reducing the production of ROS, and finally inducing the resistance of tumor cells to ferroptosis. In addition, they also observed that inhibiting the activity of CYP 1B 1 could enhance the sensitivity of tumor cells to the immunotherapeutic drug PD-1 antibody (52). The research of Sun and others supported the findings of Chen congcongcong’s team and further pointed out that melatonin could promote CYP 1B 1 overexpression, thereby inhibiting the occurrence of cell ferroptosis (53). In addition, melatonin can also exert its protective effect by inhibiting ferroptosis through regulating MDM 2 mediated ubiquitination of ACSL 4 (54).

### 3.3 Ubiquitination of GPX 4

GPX 4 plays a key role in the antioxidant defense system. With the assistance of GSH, GPX 4 can continuously decompose lipid peroxide. However, once GSH is depleted or GPX 4 activity is inhibited, intracellular Fe2+ will trigger lipid peroxidation and cause ferroptosis (55). Therefore, in order to find new drugs to treat liver cancer, we can study the effect of GPx4 transcriptional and post-translational modification on its activity in ferroptosis. It is reported that sesquiterpene lactone derivative (DMOCPTL) directly binds to GPX 4, leading to GPX 4 ubiquitination and down regulating the protein level of GPX 4, thereby inducing ferroptosis in triple negative breast cancer cells. This study first confirmed that GPX 4 ubiquitination can induce ferroptosis of tumor cells (56). Similarly, Yao et al. found that the natural product plumbagin can mediate GPX 4 ubiquitination and reduce the abundance of GPX 4 by inhibiting the expression of the deubiquitinating enzyme USP 31, resulting in ferroptosis of HCC cells (57). In addition, studies have shown that GPX 4 is a substrate of TRIM 21, and TRIM 21 overexpression mediates GPX 4 ubiquitination and degradation, increases ROS production, promotes intracellular iron accumulation, and enhances cell sensitivity to RSL 3 and erastin induced ferroptosis (58). According to the latest report, ubiquitin specific protease 8 (USP 8) can interact with GPX 4 and remove its polyubiquitination modification to maintain the stability of GPX 4 to combat colorectal tumor ferroptosis (59). However, whether it can regulate the ferroptosis sensitivity of HCC still needs further research.

### 3.4 Ubiquitination of SLC 7A 11

As mentioned earlier, the expression level of SCL 7A 11 has a direct impact on the activity of System Xc-: when the expression of SCL 7A 11 is reduced, the activity of System Xc- will decrease, resulting in reduced cystine uptake, which in turn reduces the synthesis of GSH, leading to oxidative stress mediated ferroptosis; On the contrary, overexpression of SCL 7A 11 enables tumor cells to restore redox homeostasis and enhance the resistance of tumor cells to ferroptosis, which may be one of the reasons for the resistance to chemotherapeutic drugs. Therefore, regulating the expression level of SCL 7A 11 is of great significance to overcome the drug resistance of tumor cells (26). In HCC tissues, SOCS was first verified by Chen’s team to be a specific E3 ubiquitin ligase of SLC 7A 11. The highly expressed SOCS 2 caused SLC 7A 11 to be recognized and degraded by 26S proteasome by mediating K48 ubiquitination of SLC 7A 11, eventually causing cell death and radiosensitization of hepatocellular carcinoma through ferroptosis pathway (60). Zhang et al. found that the ubiquitination degree of SLC 7A 11 can be increased by lncrna hepfal, which leads to the decrease of the stability of SLC 7A 11 and the occurrence of cell death in hepatocellular carcinoma; It was also found that lncrna hepfal could increase the sensitivity of erastin induced ferroptosis by regulating mtorc 1 (61). Notably, Xiao et al. found that SLC 7A 11-As 1 enhanced the stability of SLC 7A 11 protein by binding to the 3 ‘UTR region of SLC 7A 11 mRNA, thereby resisting erastin induced ferroptosis and promoting HCC cell growth (62). Knockdown of dazap 1 gene by siRNA technology can directly inhibit the malignant behavior of liver cancer cells. However, further studies showed that dazap 1 interacted with the 3 ′ UTR (untranslated region) of SLC 7A 11 mRNA and positively regulated its stability, revealing the multiple mechanisms of DAZAP 1 in the development of liver cancer (63). Ubiquitin specific peptidase 20 (USP 20) maintains the stability of SLC 7A 11 protein by removing K48 linked polyubiquitination of SLC 7A 11 protein at K30 and K37, inhibits the occurrence of ferroptosis in liver cancer, and increases the tolerance to oxaliplatin (64). In addition, MSC exo induced SLC 7A 11 protein expression in mesenchymal stem cells was accompanied by an increase in CD44 and OTUB 1. The abnormal expression of ubiquitinated SLC 7A 11 triggered by CCl4 can be rescued by otub1 mediated deubiquitination, thereby enhancing the stability of SLC 7A 11, activating System Xc-, and alleviating CCl4 induced hepatocyte ferroptosis (65). In summary, these findings provide new research ideas for the treatment of hepatocellular carcinoma and help to find new potential targeted therapeutic sites.

### 3.5 Ubiquitination of FSP 1

Previous studies have shown that FSP 1 is one of the important regulatory molecules involved in ferroptosis. The expression and function of FSP 1 are finely regulated by a variety of upstream factors, including transcription factors and non coding RNA molecules. In addition, FSP 1 also undergoes a series of epigenetic modifications, such as protein ubiquitination and methylation, which affect its stability and protein activity, and then affect tumor ferroptosis. It has been reported that in HCC, long noncoding RNA lncFAL can competitively bind to FSP 1, prevent FSP 1 from being degraded by TRIM 69 mediated ubiquitination, and reduce ferroptosis sensitivity; It was also demonstrated that HDLBP combined with lncFAL can stabilize its expression (66). It provides a potential target for the treatment strategy of HCC patients with high expression of HDLBP or lncFAL.

Sorafenib enhanced the ferroptosis sensitivity of HCC by activating ERK signaling pathway to promote the ubiquitination and subsequent protein degradation of FSP 1 by TRIM 54. By inhibiting TRIM 54, the sorafenib induced ferroptosis effect can be reversed. In addition, FSP 1 expression level is positively correlated with tumor development *in vivo* and may reduce the sensitivity of HCC cells to sorafenib (67). These findings revealed the potential mechanism of sorafenib in HCC treatment and provided a new perspective for future treatment strategies.

### 3.6 Ubiquitination of NRF 2

Studies have shown that NRF 2 can not only promote the synthesis and regeneration of GSH, maintain the level of GSH in HCC and reduce its consumption to play an antioxidant role; Moreover, it can counteract sorafenib induced ferroptosis by activating the expression of downstream antioxidant gene HO-1 (heme oxygenase-1) via p62-Keap 1-NRF 2 pathway (35). NRF 2 regulates ubiquitination and proteasomal degradation in antioxidant responses by promoting gene expression of antioxidant and detoxification enzymes in combination with antioxidant stress elements (ARE), enhancing the antioxidant effect of cells (68). P62 is an autophagy related protein that can interact with the NRF 2 binding site on Keap1 (kelch like ECH related protein 1). As an adaptor protein of cullin-3 ubiquitin ligase of NRF 2, Keap1 is involved in the ubiquitination and degradation of NRF 2 (69). However, when autophagy is impaired or p62 is overproduced, the accumulation of p62 will compete with the interaction between NRF 2 and Keap1. This competitive inhibition prevents the degradation of NRF 2. NRF 2 activates the transcription of antioxidant enzymes by interacting with transcriptional coactivators, thereby activating the antioxidant effect and inhibiting the ferroptosis function of liver cancer (35, 70). In addition, the p62-Keap 1-NRF 2 signaling pathway is inhibited by TRIM 21, a ubiquitin E3 ligase, through the release of p62 oligomerization at the K7 site in the PB1 domain of ubiquitinated p62 (71). CISD 2 knockdown can promote the degradation of p62, enhance the binding affinity between Keap 1 and NRF 2, and then promote ubiquitination and degradation of NRF 2, resulting in oxidative stress and ferroptosis (72). In conclusion, activating p62-Keap 1-NRF 2 signaling pathway can reduce the sensitivity of tumor cells to ferroptosis, providing a new strategy for the treatment of liver cancer. In addition, the latest research found that inhibiting the expression of apurinic/apyrimidinic endonuclease 1 (APE 1) would lead to the ubiquitination of NRF 2. The downregulation of NRF 2 inhibited the expression of SLC 7A 11 and GPX 4, caused peroxidation, and triggered ferroptosis in HCC cells (73). This revealed a new role and mechanism of APE 1 in regulating ferroptosis. See **Table 1**.

**Table 1:** Ubiquitination modification of HCC ferroptosis related molecular proteins.

HCC Ferroptosis pathway	Target	Ubiquitinase/deubiquitinase	Function	Effect on HCC ferroptosis	Reference
Iron metabolism pathway	IRP	SCF FBXL5	Promote the ubiquitination and degradation of IRP	+	(44)
	TFRC	βTrCP	Trib 2 promotes the ubiquitination and degradation of TFRC through E3 β trcp	−	(47)
		NEDD 4L	Nedd 4L promotes the ubiquitination and degradation of TFRC	−	(50)
Peroxidation of unsaturated phosphate	ACSL 4	FBXO 10	Promote the ubiquitination and degradation of ACSL 4	−	(52)
		MDM 2	Promote the ubiquitination and degradation of ACSL 4	−	(54)
System Xc-	GPX 4	USP 31	Plumbagin inhibits USP 31 expression and promotes GPX 4 ubiquitination and degradation	+	(57)
		TRIM 21	Promote the ubiquitination and degradation of GPX 4	+	(58)
	SLC 7A 11	SOCS	Mediates K48 ubiquitination of SLC 7A 11, causing SLC 7A 11 to be recognized and degraded by the 26S proteasome	+	(60)
		USP 20	The stability of SLC 7A 11 protein was maintained by removing K48 ubiquitination of SLC 7A 11 protein at K30 and K37	−	(64)
		OTUB1	Deubiquitinating enzyme otub1 enhances the stability of SLC 7A 11	−	(65)
		TRIM26	TRIM26 mediates the ubiquitination and degradation of SLC 7A 11	+	(75)
		RNF182	PCDHB14 promoted rnf182 mediated p65 ubiquitination, blocked p65 binding to the SLC 7A 11 promoter, and SLC 7A 11 expression decreased	+	(76)
NADPH-FSP 1-CoQ Defense pathway	FSP 1	TRIM 69	Long noncoding RNA lncFAL competitively binds FSP 1 and prevents FSP 1 from being degraded by TRIM 69 mediated ubiquitination	−	(66)
		TRIM 54	Sorafenib promotes the ubiquitination and degradation of FSP 1 by TRIM 54	+	(67)
Other ways of ferroptosis	NRF 2	TRIM 21	TRIM 21 inhibits the p62-Keap 1-NRF 2 signaling pathway by ubiquitinating the K7 site in the PB1 domain of p62	+	(71)

+, Promote; −, Suppression.

### 3.7 Ubiquitination of other regulators

Among the many members of ubiquitinating enzymes, studies have pointed out that a specific type of methyltransferase (L5) plays a role in regulating the expression of deubiquitinating enzyme USP 5. L5 can block the ubiquitination of c-Myc protein linked by K48 chain by affecting the translation process of USP 5, which is a protein degradation related modification. In this way, L5 indirectly changed the metabolic pathway of glucose, leading to the further development of liver cancer (74). In the mouse model induced by CCl4, Zhu et al. found that E3 ubiquitin ligase TRIM26 mediates SLC 7A 11 ubiquitination, targets this protein for proteasomal degradation, and causes hepatic stellate cells to undergo ferroptosis, thereby alleviating the process of liver fibrosis (75). Therefore, exploring substances that can activate TRIM26 may become an effective method for the treatment of early-stage hepatocellular carcinoma. Interestingly, PCDHB 14 accelerates the downregulation of p65 levels by promoting p65 ubiquitination mediated by the E3 ubiquitin ligase RNF 182, and then blocks the binding of p65 to the SLC 7A 11 promoter. The decreased expression of SLC 7A 11 enhances ferroptosis sensitivity. In addition, p53, as an important tumor suppressor protein, can inhibit the proliferation of tumor cells and promote ferroptosis by inducing the expression of PCDHB 14, promoting the downregulation of SLC 7A 11 levels (76). These studies show that there are still many unknown regulators that affect ferroptosis by regulating SLC 7A 11 levels directly or indirectly. In the treatment study of HCC, QSQX 1 (thiol oxidase 1) was found by Zhou et al. To induce ferroptosis by promoting EGFR ubiquitination and degradation, while inhibiting the activation of NRF 2, suggesting that QSQX 1 may become a new target for the treatment of hepatocellular carcinoma (77).

## 4 Targeted regulation of ubiquitination of ferroptosis related molecules in the treatment of liver cancer

In recent years, with researchers’ in-depth understanding of the molecular biological mechanism of HCC, in addition to traditional treatment methods, a variety of new treatment methods have shown great potential in the diagnosis and treatment process. As mentioned above, ubiquitination can affect the stability and activity of many molecules in the ferroptosis pathway and mediate cancer cell death. It can be speculated that the treatment of cancer can be achieved by targeting ubiquitination to induce ferroptosis in HCC. Studies have shown that plumbagin, a bioactive compound extracted from the root of the Chinese herbal medicine plumbago alba, can down regulate GPX 4 levels by mediating GPX 4 ubiquitination, resulting in increased sensitivity of HCC cells to ferroptosis; While overexpression of GPx4 inhibited the apoptosis of HCC cells induced by plumbagin (59). In addition, proteasome deubiquitinase (DUB) inhibition was shown to trigger proteasome dysfunction and promote apoptosis in tumor cells. Li et al. found that palladium pyridine complex (PdPT), as an inhibitor of broad-spectrum DUBs, can not only lead to apoptosis, but also inhibit the proliferation and invasion of liver cancer cells by inducing ferroptosis through deubiquitination and degradation of GPX 4. This indicates that DUB is a potential target for the development of antitumor drugs (78).

It has been reported that by using proteolysis targeting chimeras (PROTACs) technology, such as dGPX 4, a molecule designed based on PROTACs, it can down regulate GPX 4 and induce ferroptosis, which may become a new method for the treatment of cancer. And the E3 ligase in protac molecule can also bind to the mutated protein, trigger its degradation, and exert its anti-drug resistance ability (79). It is worth noting that the application of this technology may be limited by the ability of genetic manipulation (80). Therefore, the latest research shows that artificially designed long non coding RNA (alncRNA) may become an effective alternative strategy, which promotes the ubiquitination and degradation of target proteins through the synergistic effect with E3 ubiquitin ligase Dzip3, which may become a new strategy for the treatment of liver cancer (81).

Besides cancer, the regulation of ubiquitination on ferroptosis also plays a role in the treatment of other diseases. As a deubiquitinating enzyme, mysm1 regulates trim 21 stability and mediates doxorubicin (DOX) - induced ferroptosis in cardiomyocytes, suggesting that cardiomyocyte specific knockdown of mysm1 may be an attractive therapeutic strategy for this disease (82). The latest research found that Hyperoside, a bioactive compound extracted from traditional Chinese medicine, promotes the translocation of Nrf2 to the nucleus by inhibiting ubiquitin mediated degradation of Nrf2, resulting in the up regulation of the expression of anti ferroptosis genes, and inhibiting ferroptosis in the treatment of sepsis induced acute lung injury (83). Therefore, it is a promising strategy to develop traditional Chinese medicine to regulate the ubiquitination of ferroptosis related molecules in the treatment of liver cancer in the future.

## 5 Conclusion

Ferroptosis is an important pathway of tumor cell death. Previous studies have shown that ferroptosis combined with other treatment methods is effective in the treatment of liver cancer; And it can effectively promote or inhibit ferroptosis by targeting the ubiquitination level of molecules related to ferroptosis pathway, which is a promising strategy for the treatment of liver cancer. In the future, we can enhance the sensitivity of liver cancer cells to ferroptosis by developing small molecule inhibitors or activators that specifically regulate E3 ubiquitin ligases, such as palladium pyridine complex (PdPT), an inhibitor of broad-spectrum DUBs, and affecting the ubiquitination status of ferroptosis related proteins. In general, ubiquitination of key factors of ferroptosis provides a new research direction for disease treatment. However, most of the research in this field is still at the cellular and animal levels, and more experimental research is still needed to turn new treatment options into applications to solve clinical problems.
